# Colombian Rasch validation of KIDSCREEN-27 quality of life questionnaire

**DOI:** 10.1186/s12955-016-0472-0

**Published:** 2016-05-04

**Authors:** Claudia-Marcela Vélez, Luz-Helena Lugo-Agudelo, Gilma-Norela Hernández-Herrera, Héctor-Iván García-García

**Affiliations:** Group of Clinical Epidemiology (GRAEPIC), School of Medicine, University of Antioquia, Medellín, Colombia; Department of Clinical Epidemiology and Biostatistics, McMaster University, 1280, Main, Hamilton, Ontario Canada; Academic Group of Clinical Epidemiology (GRAEPIC) and Group Health Rehabilitation, School of Medicine, University of Antioquia, Carrera 51 D # 62-29, Medellín, Colombia

**Keywords:** Health-related quality of life, KIDSCREEN, Psychometric properties, Rasch analysis, Calidad de vida relacionada con la salud, Kidscreen, Psicometría, análisis Rasch

## Abstract

**Background:**

The family of KIDSCREEN instruments is the only one with trans-cultural adaptation and validation in Colombia. These validations have been performed from the classical test theory approach, which has evidenced satisfactory psychometric properties. The aim of this study was to evaluate psychometric properties of KIDSCREEN-27 children and parent-proxy versions, through Rasch analysis.

**Methods:**

The participants in the present study were two different sets of populations, 321 kids with a mean age of 12.3 (SD 2.6), 41 % 8 to 11 years old and 59 % 12 to 18 years old; and 1150 parent-proxy with an average age of 45.5 (SD 18.9). Psychometric properties were assessed using the partial credits model in the Rasch approach. Unidimensionality, fitting of person and item, response form, and differential item functioning (DIF) were measured.

**Results:**

The Infit MNSQ in child self-reported version that ranges between 0.71–1.76, and 0.69–1.31 in the parent-proxy version. Scores gathered on Likert forms of 5-response options, person separation was 2.08 for child self-reported version and 2.40 for parent-proxy; reliability was 0.81 and 0.85, respectively. Items reliability was 0.99 on both versions, with separations of 11.92 for child self-reported and 10.83 for parent-proxy. There was not DIF according to the variables sex and age but was present according to socioeconomic status.

**Conclusion:**

There was a good fit for items and individuals to the Rasch model. Item separation was adecuate, and person separation improved when the response form was re-codified to four options. The presence of DIF according to socioeconomic status implies a scale’s bias in the measure of HRQoL of Colombian children.

## Resumen en español

**Introducción:**

La familia de instrumentos de medición de calidad de vida en niños KIDSCREEN, es la única que cuenta con adaptación transcultural y validación por teoría clásica del test, la cual ha evidenciado propiedades psicométricas satisfactorias. El objetivo de este estudio fue evaluar por análisis Rasch las propiedades psicométricas de KIDSCREEN-27 en sus versiones niños y padres.

**Métodos:**

Participaron 321 niños con una edad promedio de 12.3 años (DE 2.6), 41 % entre 8 y 11 años y 59 % entre 12 y 18 años de edad; y 1150 padres o acudientes con una edad promedio de 40 años (rango 18 a 79). El análisis estadístico se hizo por el modelo de Créditos Parciales de la aproximación Rasch. Se evaluó la unidimensionalidad, confiabilidad de personas e ítems, formato de respuesta, y funcionamiento diferencial de los ítems (DIF).

**Resultados:**

El Infit MNSQ de las respuestas de niños estuvo entre 0.71–1.76, y el de padres osciló entre 0.69–1.31. Con la puntuación recogida en un formato Likert de 5 opciones de respuesta, se observa que la separación de las personas es de 2.08 en niños y de 2,40 en padres, mientras que la confiabilidad fue de 0.81 para niños y de 0,85 para padres. En relación con la separación de los ítems, la confiabilidad fue de 0,99 en ambas versiones, pero con separación de 11.92 y 10,83, respectivamente. No se detectó DIF al comparar grupos de acuerdo con las variables sexo y edad, pero estuvo presente al comparar por condición socioeconómica.

**Conclusión:**

Hubo un buen ajuste de los ítems y las personas al modelo Rasch, tanto en los cuestionarios diligenciados por padres como por niños. La separación de los ítems fue muy buena, y la separación de las personas mejoró cuando se recodificó el formato de respuesta a solo 4 opciones. La presencia de DIF de acuerdo a la condición socioeconómica implica un posible sesgo en la medición de la calidad de vida en niños colombianos.

## Background

Research and assessment of Health-related quality of life (HRQoL) on children have experienced substantial progress during the last few years. Some instruments are disease-specific and others are generic. In the latter group, the family of KIDSCREEN questionnaires is the most widely used [1]. Amongst its advantages are their multidimensional construct based on the World Health Organization’s definitions of health and quality of life [2], and their simultaneously development in 13 European countries, which improves their trans-cultural applicability. Previous examinations have showed that KIDSCREEN-27 exhibits good psychometric properties in terms of Rasch measurement, reliability and validity of its scores [3–8]. KIDSCREEN questionnaires were developed for self-perceived health measures concerning physical, emotional, mental and social well-being in children and adolescents aged 8 to 18 years. There are three versions: KIDSCREEN-52, KIDSCREEN-27, and KIDSCREEN-10, available for children/adolescents self-report and for parents-proxies.

Traditionally, the construct validity of scales has been developed from the Classical Test Theory approach (CTT). However, the availability of new statistic software enables the use of more powerful and discriminative techniques like Rash analysis [9, 10]. CTT and Rasch approaches provide numerous statistical techniques to examine the psychometric quality of the items and to detect weak or biased items, but with different theoretical assumptions. CTT is based on the Spearman linear model which proposes a linear relationship between observed and expected scores and assumes that score’s variations reflect differences in measured traits among different subjects [11]. Item Response Theory (IRT) gathers a set of probabilistic models, among which Rasch analysis is the most widely used and accepted [11]. It does not assume that each item is equally difficult, and therefore, Rasch incorporates the idea that the probability of a correct response to an item is a mathematical function of person and item parameters [12]. This mathematical relationship instead of a linear function is a logistic function of the relative difference between item difficulty and respondent trait level [13].

Some advantages of Rasch analysis are that: 1) allows parametric statistical analysis, which is an advantage over CTT given that facilitates linear regression analysis in different epidemiological studies; 2) provides accurate estimations of individual measurement errors, and identifies biased and inappropriate items [14]; and 3) estimates both item and person statistics independently of the current sample of items and persons respectively [15] allowing more versatile applications of the measurements in different situations and with different populations.

The measurement of the scale properties by Rasch analysis include the assessment of the fitting of data to the model [16], and the measurement of differential item functioning (DIF), which occurs when the likelihood of answering an item correctly by individuals with the same trait level varies according to their age or sociocultural group [17]. Increasingly, Rasch analysis has demonstrated satisfactory psychometric properties of KIDSCREEN-27, as unidimensionality, the fit of person and items, and absence of DIF when comparing children and adolescentes, in both healthy and ill kids [10, 18, 19].

In Colombia, the validation of HRQoL questionnaires for children and adolescents is incipient, only the generic questionnaires KIDSCREEN-27 and recently PedsQL 4.0 (unpublished results) have CTT validations, both showing satisfactory internal consistency, content and construct validity [20, 21]. Our research team validated KIDSCREEN-27 child self-reported version in 2007 [20], and the KIDSCREEN-27 parent-proxy version in 2012 [21]. The aim of this article was to improve the information about the psychometric properties of KIDSCREEN-27 child and parent-proxy versions using the Rasch measurement model, contrasting the results with those obtained from the CTT analysis. Our hypothesis is that KIDSCREEN-27 has appropriated psychometric properties to measure HRQoL in children and adolescents in Colombia.

## Methods

### Instrument

The KIDSCREEN-27 is a questionnaire which assesses HRQoL in five dimensions: Physical Well-being (PW) has five items to valuate physical activities and health; Psychological Well-being (PsW) has seven items that examine the psychological well-being of the child/adolescent, including positive emotions and satisfaction with life; Autonomy & Parent Relations (APR) has seven items to explore the family environment and child/adolescent opportunities to perform activities in his/her spare time; Social Support & Peers (SS) has four items to obtain information about the relationship between child/adolescent and his/her peers; School Environment (SE), with four items, explores child/adolescent self-perception of cognitive capacity, concentration and social relationships at school. Responses to KIDSCREEN-27 questionnaire measure frequency (never-seldom-sometimes-often-always) or intensity (not at all-slightly-moderately-very-extremely) of the assessed attribute with the 5-options Likert scale, with a recall period of one week. Rasch scores are computed for each dimension and are transformed into *T*-values with a mean of 50 and a standard deviation of 10; higher scores indicate better HRQoL and well-being [4, 18].

### Sample

Each Colombian CTT validation of KIDSCREEN-27 estimated the sample size according to the psychometric properties reported by original validation studies [4, 20, 21], in order to compare the HRQoL between children (8–11 years old) and adolescents (12–18 years old) of high and low income families (strata 4, 5 and 6 vs. strata 1, 2 and 3 of Colombian government classification). Estimation of the sample size considered different psychometric properties: internal consistency, contents and construct validity, test-retest reliability, and parent-child agreement. The final sample included 321 children and adolescents, and 1150 parents-proxies.

### Data gathering

Children and adolescents: Data of 321 children and adolescents aged 8–18 years were gathered by three trained professionals between November 2006 and November 2007. 161 healthy kids were invited to participate in schools, and 160 ill kids were approached in hospitals (hospitalization and out-patient services) of Medellin. Questionnaires were administered in two ways; 105 were self-administered and 216 through interview. Those interviewed were principally ill kids or with some level of limitation (78 % had some level of limitation, 97 % ill kids).

Parent: Parents-proxies were approached when they assisted for receiving the school reports cards between October 2011 and March 2012. Of thirteen schools invited to participate, four public and three private schools took part. Due to the low education status of some individuals, questionnaires were administered in two ways, 1105 through self-administration and 45 required the assistance of research team members. Each child or adolescent had only one parent answering the questionnaire,

Besides schools approval, informed consent was secured from families along with acquiescence from children and adolescents. Both research studies were approved by the Ethics Committee of the School of Medicine, University of Antioquia.

### Statistical analysis

Partial Credits Model is a member of the family of latent trait models in Rasch which share the property of parameter ‘separability’ and ‘specifically objective’ comparisons of persons and items. It is applicable to scales with polytomous items with ordered categories such as Likert scales, allowing different thresholds for different items [22, 23]. The Rasch analysis included the measurement of total model fit, unidimensionality, person and item reliability, response form (maps and thresholds) and DIF.

One of the Rasch model assumptions is that items address a single construct with a little overlap between items [22]. To assess whether such assumption is met, principal components analysis is implemented. This study assessed the explained variance, considering acceptable if it was greater than fifty percent (>50 %) [24, 25]. To define non-compliance with the one-dimensional assumption, a “secondary dimension” on non-explained variance should have at least three items (*eigenvalues* of the first contrast >3) [26] and correlations among residuals greater than 0.2 [26].

#### Validity of contents

Mean Square residuals (MNSQ) were used to assess the fitting of data to Rasch model. *Infit* and *outfit* indexes were estimated, the first one indicates adjustment between expected and observed average values while the second one considers unexpected answers from individuals [27]. When the observed data fits the model, MNSQ values are close to 1, the fitting is considered acceptable if values are between 0.7 and 1.4 [4, 28–31].

#### Internal consistency

Separation of person and items was measured assessing the power of the measurement among respondents with different trait levels and items with different difficulty. In other words, if response options to an item are: never, seldom, sometimes, often, and always; the scale should properly separate those answering *never* from those answering *seldom* because they are theoretically different at the trait level. Separation of items and individuals must be at least three standard errors, and such measure correlates with reliability, which should be greater than 0.7 [27, 32].

#### The Response Format

Distinctive items curves allow spotting disorderly thresholds, occurring when individuals do not use response categories consistently with the measured trait level. When the disorder was detected at the threshold, score re-codification was considered. Initially, the fit to the model was evaluated according to five categories of response proposed by the developers (1 = never/not at all, 2 = seldom/slightly, 3 = sometimes/moderately, 4 = often/very, 5 = always/extremely). Subsequently, after the new analysis, categories were merged and reduced to 4, 3 and 2 response options, in order to assess items separation and scale’s ability to discriminate properly and organize individuals based on their responses.

#### Differential Item Functioning (DIF)

Potential bias in items might be identified when individuals respond differently to the item in different groups of the sample, despite the same measured trait level. Each item was examined in order to detect DIF based on four variables: sex, age (8–11 years and 12–18 years), socio-economic status (low and high), and health status (healthy and ill). A difference larger than 0.5 in terms of difficulty of the items among groups was considered positive for DIF [29]. The Welch’s t test was used to assess the difference, also a Mantel and Hanzel test to evaluate observed and expected DIF values. The test was considered statistically significant if the *p* value was ≤ 0.005 after Bonferroni correction [29].

Statistical analysis was performed using the Winsteps 3.7.10 software (Beaverton, Oregon: Winsteps.com) [26].

## Results

### Population description

The final sample included 321 children and adolescents, and 1150 parents-proxies. The mean age of the participants in the KIDSCREEN-27 child version validation was 12.3 (SD 2.6), 41 % were 8 to 11 years age and 59 % were 12 to 18 years age; 43 % were women and 57 % attended public schools. Diagnoses of the 160 ill kids were: diseases of cardiopulmonary, digestive, respiratory and urinary systems (30 %); musculoskeletal system (25.6 %); congenital malformations (13.1 %); central and peripheral nervous system (12.5 %); infections (8.8 %); neoplasms (5 %), and neurological and sense organs’ diseases (5 %). Children and adolescents with limitations mainly had impairment to walk and move (51 %); to communicate (25.5 %); to exercise (7.8 %); to use the hand(s) and arm(s) (7.8 %); to learn and apply knowledge (2 %); to see (3.9 %), and related to the genitourinary function (2 %).

Among the 1150 parents-proxies, the average age was 45.5 (SD 18.9), 80 % were women, and 13 % with five or fewer years of literacy (80 % less than eleven years of literacy). Out of the total sample, 1002 (87 %) were parents and 148 (12.9 %) were other relatives. The average age of their offspring was 12.9 (SD 2.7); they were mainly teenagers from 12 to 18 years (70.4 %), and a great proportion of them were males (56.8 %) from public schools (88 %) (Table 1).

**Table 1:** Demographic characteristics of children based on age group

Variable	KIDSCREEN-27 child self-report			KIDSCREEN-27 parent-proxy report		
	Children	Adolescents	Total	Children	Adolescents	Total
	(*n* = 131)	(*n* = 190)	(*n* = 321)	(*n* = 340)	(*n* = 810)	(*n* = 1150)
	# (%)	# (%)	# (%)	# (%)	# (%)	# (%)
Men	78 (59.5)	105 (56.2)	183 (57)	191 (56.2)	462 (57.0)	653 (56.8)
Women	53 (40.5)	85 (43.8)	138 (43)	149 (43.8)	348 (43.0)	497 (43.2)
Public school	81 (61.8)	103 (54.2)	184 (57.3)	274 (80.6)	738 (91.1)	1012 (88,0)
Private school	50 (38.2)	87 (45.8)	137 (42.7)	66 (19.4)	72 (8.9)	138 (12.0)
Socioeconomic status						
Low	69 (52.7)	80 (42.1)	149 (46.4)	194 (57.1)	399 (49.3)	593 (51.6)
Medium	44 (33.6)	60 (31.6)	104 (32.4)	104 (30.6)	345 (42.6)	449 (39.0)
High	18 (13.7)	50 (26.3)	68 (21.2)	42 (12.4)	66 (8.1)	108 (9.4)
Health condition						
Healthy	67 (51.1)	94 (49.5)	161 (50.1)	274 (80.6)	672 (83.0)	946 (82.3)
Ill	64 (48.9)	96 (50.5)	160 (49.9)	66 (19.4)	138 (17.0)	204 (17.7)

#### Responsiveness

The response rate for both KIDSCREEN-27 versions was 99 % with a completion rate of 97 % in children and 99 % in parent-proxy. The imputation of missing data was performed according to KIDSCREEN group recommendations; this is, with the average value of the dimension when the person has answered at least 75 % of the items [33].

### The quality of Life

On the validation of KIDSCREEN-27 child version, HRQoL was lower in all dimensions for children and adolescents that were ill, excepting the PsW dimension in which the scores were similar (*p* < 0.05). Men scored higher than women, excepting on the SS and APR dimensions (*p* > 0.05). HRQoL on high socioeconomic staus was higher than on those lower (*p* < 0.05). Scores were also better for children from private schools over those from public schools, being statistically significant for the PsW, APR, and SS dimensions (Table 2).

**Table 2:** Scores for each child self-report dimension based on sex, age, socioeconomic status, type of school and health status

Dimension	Total n = 321	Sex		Socioeconomic status			Age		Health status		Type of school	
		Men	Women	Low	Middle	High	Children	Adolescents	Ill	Healthy	Public	Private
		*n* = 183	*n* = 138	*n* = 149	*n* = 104	*n* = 68	*n* = 131	*n* = 190	*n* = 160	*n* = 161	*n* = 184	*n* = 137
Physical well-being												
Mean:	65,1	67.3	64.4	64.9	66.9	67.6	69.2	64.0	59.0	73.1	65.2	67.3
(sd)	18.6	18.1	19,2	19.2	18.2	18.1	19.2	17.9	16.40	18.0	19.3	17.6
Psychological well-being												
Mean:	77.3	78.4	75.8	74.8	79.1	76.5	79.1	76.0	75.3	79.2	75.3	80.0
(sd)	15.2	15.3	14.9	15.6	14.8	14.2	14.2	15.7	14.2	15.9	16.1	13.4
Autonomy & parent relations												
Mean:	73.5	72.7	74.5	71.0	73.1	79.3	74.2	73.0	69.0	77.9	70.2	77.8
(sd)	17.2	18.0	16.2	17.5	17.0	16.0	16.6	17.7	14.1	18.8	17.6	15.7
Social support & peers												
Mean:	75.8	74.2	77.8	72.3	75.7	78.5	76.0	75.6	67.5	84.0	79.9	79.9
(sd)	23.4	22.9	24.2	23.1	24.5	21.0	23.6	23.5	25.3	18.0	23.6	22,7
School environment												
Mean:	75.0	75.5	74.2	74.1	74.2	78.0	77.6	73.1	69.6	80.3	72.4	78.3
(sd)	19.7	18.8	20.9	18.0	22.2	19.5	22.4	17.5	22.0	15.6	20.0	19.0

In the validation of KIDSCREEN-27 parent-proxy version, women scored lower than men (*p* < 0.05), except for the SE dimension (*p* > 0.05). Adolescents scored lower than children, except for the SS dimension (*p* < 0.05). HRQoL was also better in all dimensions when the socioeconomic status was higher (*p* < 0.05); children attended private schools (*p* < 0.05), and they were healthy (*p* > 0.05 in PsW, APR, SE).

#### Items difficulty

In KIDSCREEN-27 children version difficulty range between −0.71 (“have your parents treated you fairly?”) to 1.99 (“have you felt so bad that you didn't want to do anything?”). In the parent-proxy version, difficulty measures were between −0.71 (“has your child felt treated fairly?”) to 0.92 (“has your child had enough money as his/her friends?”) (Table 3).

**Table 3:** Item difficulty, infit, and outfit MNSQ statistics for each item of the KIDSCREEN-27

Item	KIDSCREEN-27 child self-report				KIDSCREEN-27 parent proxy-report			
	Difficulty	Model S.E.	INFIT MNSQ	OUTFIT MNSQ	Difficulty	Model S.E.	INFIT MNSQ	OUTFIT MNSQ
1 How is in general your health	1.08	.06	1.69	1.72	.12	.04	1.07	1.16
2 Have you felt fit and well	-.02	.06	.95	.96	.29	.03	.85	.87
3 Have you been physically active	.50	.05	.91	.92	.50	.03	1.27	1.25
4 Have you been able to run	.48	.05	.92	.95	.19	.04	1.04	.99
5 Have you felt full of energy	-.50	.08	.87	.85	-.39	.04	.79	.74
6 Have you enjoyed your life	-.48	.07	.82	.80	-.20	.04	.78	.79
7 Have you been in a good mood	-.60	.07	.94	.92	.00	.04	.69	.69
8 Have you had fun	-.53	.07	.79	.76	-.39	.04	.69	.67
9 Have you felt sad	1.82	.07	1.58	1.67	.45	.03	.81	.89
10 Have you felt so bad that you didn't want to do anything	1.99	.07	1.67	2.07	-.17	.04	1.10	1.17
11 Have you felt lonely	1.91	.06	1.76	2.56	-.03	.04	1.14	1.16
12 Have you been happy	-.46	.07	.99	.90	-.56	.04	1.23	1.49
13 Have you had time to yourself	-.38	.07	.87	.79	-.67	.04	.92	.91
14 Have you had free time	.10	.06	.92	.95	-.21	.04	.84	.86
15 Have your parents had enough time for you	-.63	.07	1.12	1.01	-.06	.04	.89	.87
16 Have your parents treated fairly	-.71	.07	1.17	1.16	-.71	.05	.93	.85
17 Have you been able to talk to your parents	-.62	.06	1.11	1.01	-.56	.04	1.14	1.03
18 Have you had enough money as your friends	-.21	.05	.89	.89	.92	.03	1.31	1.37
19 Have you had enough money for your expenses	-.30	.05	.84	.84	.57	.03	1.18	1.22
20 Have you spent time with your friends	-.54	.06	.71	.65	.15	.04	1.02	1.04
21 Have you had fun with your friends	-.62	.06	.74	.66	-.07	.04	1.08	1.04
22 Have you and your friends helped each other	-.53	.06	.74	.70	.24	.04	.99	1.02
23 Have you been able to rely on your friends	-.43	.05	.88	.91	.79	.03	1.31	1.40
24 Have you been happy at school	-.07	.06	.76	.73	-.06	.04	1.04	1.00
25 Have you got on well at school	.09	.06	.85	.85	.36	.03	1.12	1.21
26 Have you been able to pay attention	-.08	.07	.81	.77	.05	.04	.88	.92
27 Have you got along well with your teachers	-.27	.07	.94	.98	-.55	.04	.99	1.02

#### Internal scale validity

In the KIDSCREEN-27 child self-report version all items showed Infit MNSQ values within the range (0.7–1.40) except for four items: “How is in general your health?” (1.69) within the dimension Physical Well-being, “Have you felt sad?” (1.58), “Have you felt so bad that you didn't want to do anything?” (1.67), “Have you felt lonely?” (1.76) within Psychological Well-being (Table 3). In the KIDSCREEN-27 parent-proxy version all items showed Infit MNSQ values within the range (0.7–1.40) except for: “Has your child been in a good mood?” (0.69), and “Has your child had fun?” (0.69).

#### Unidimensionality

The explained variance within KIDSCREEN-27 child self-reported version was 42.3 %; the unexplained variance in 1^st^ contrast was 6.4 %. For KIDSCREEN-27 parent-proxy version, the explained variance was 46.6 %, and the unexplained variance in 1^st^ contrast was 7.3 %.

#### Separation

With the scores gathered on a 5 response-options Likert form, the separation observed between individuals was 2.08 for the KIDSCREEN-27 children and 2.40 for the parents-proxies, while reliability was 0.81 and 0.85, respectively. In regard to item separation, reliability was 0.99 on both versions, but with separation of 11.92 for children and 10.83 for parents-proxies.

**Fig. 1:**
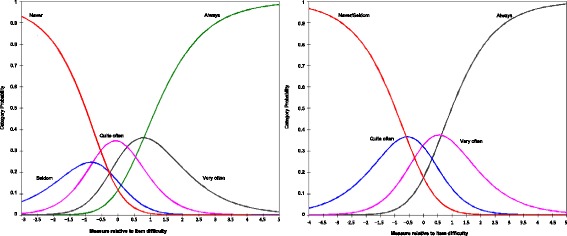
Category probability curves of five and four response categories of the KIDSCREEN-27 on item 14. On the left side are presented the category probability curves of KIDSCREEN-27 child self-report with five response categories, on the right side are presented the category probability curves of four response categories. Item 14 “Have you been able to do the things that you want to do in your free time”

When response forms were changed, the range of items separation stayed 8.03 to 10.97, and reliability moved from 0.98 to 0.99; while person separation stayed between 1.52 and 2.54, and reliability between 0.70 and 0.87. In comparison with the original response form 12345 (1 = never/not at all, 2 = seldom/slightly, 3 = sometimes/moderately, 4 = often/very, 5 = always/extremely), the response form 11234 (brings together never/not at all and seldom/slightly options, and maintaining sometimes/moderately, often/very, and always/extremely options) reported the best explained variance in both children and parents-proxies versions (45.7 and 47.31 % respectively), and improved item and person separations (Fig. 1), (Table 4).

**Table 4:** Reliability, separation indices, and Outfit MNSQ range, based on different response form models

Scoring Models	KIDSCREEN-27 version	Person		Item	
		Reliability (separation)	Outfit MNSQ range	Reliability (separation)	Outfit MNSQ range
Five categories (12345)	Parent proxy-report	0.85 (2,40)	0.14;7.55	0.99 (10.83)	0.67;1.49
	Child self-report	0.81 (2.08)	0.23;5.81	0.99 (11.92)	0.65;2.56
Four categories (11234)	Parent proxy-report	0.86 (2.45)	0.15;6.06	0.99 (10.97)	0.67;1.36
	Child self-report	0.85 (2.37)	0.27;8.54	0.99 (11.49)	0.69;3.10
Two categories (11112)	Parent proxy-report	0.84 (2.30)	0.66;4.54	0.99 (9.15)	0.76;1.51
	Child self-report	0.82 (2.16)	0.28;9.90	0.98 (7.27)	0.80;8.37
Three categories (11233)	Parent proxy-report	0.70 (1.52)	0.14;7.52	0.99 (9.18)	0.56;1.37
	Child self-report	0.75 (1.75)	0.30;9.52	0.99 (10.19)	0.49;3.08
Three categories (11223)	Parent proxy-report	0.86 (2.50)	0.10;5.63	0.99 (10.02)	0.70;1.34
	Child self-report	0.83 (2.17)	0.16;7.74	0.99 (11.12)	0.77;2.57

#### Differential Item Functioning (DIF)

DIF was not detected in KIDSCREEN-27 child self-reported version when comparing children and adolescents, nor was in girls and boys; it was identified for the item “How is in general your health?” (0.91) when comparing ill and healthy kids, and for questions “Have you felt sad?” (−0.58), “Have you felt so bad that you didn't want to do anything?” (−0.82) and “Have you felt lonely?” (0.59) when comparing participants from high and low socioeconomic status. In KIDSCREEN-27 parent-proxy version DIF was detected when comparing children and adolescents in question “Has your child been physically active?” (0.57), when comparing healthy and ill kids in item “In general, how would your child rate her/his health?” (0.66), and for questions “Has your child been happy?” (−0.56), “Has your child had enough money as your friends?” (0.57) and “Has your child had enough money for your expenses?” (0.70) when comparing participants from high and low socioeconomic status (Table 5).

**Table 5:** Items positive for Differential Item Functioning (DIF), based on sex, age, socioeconomic status, and health status

Version:	Sex	Age	Socioeconomic status	Health status
	Female/male	8–11/12–18	high/low	Healthy/Ill
	Item (DIF)	Item (DIF)	Item (DIF)	Item (DIF)
Parents	None	3 (0.57)**	12 (−0.56)*	1 (0.66)*
			18 (0.57)*	
			19 (0.70)*	
Children	None	None	9 (−0.58)*	1 (0.91)*
			10 (−0.82)*	
			11 (0.59)*	

* *p* < 0,0001 (after Bonferroni correction)

** *p* < 0,001 (after Bonferroni correction)

## Discussion

In this study, we evaluated the psychometric properties of KIDSCREEN-27 by Rasch analysis in two different samples (children/adolescents and parents/proxies). Overall, the results were satisfactory, with high response rates, good reliability, and acceptable item goodness-of fit in 23 of 27 items.

The aim of this study was to complement our previous classical test theory validations with a modern technique such as Rasch analysis. The Rasch analysis is a psychometric approach allowing identification of aspects relative to measurement not easily detected by traditional analysis, such as item bias and problems with the response form [13]. The Rasch model measures the intrinsic difficulty of items independently of the trait level of measured individuals. The combination of both classical and modern methods contributes to having more and better information about the psychometric properties of this scale.

This study found that in KIDSCREEN-27 child self-reported, items “How is in general your health?”, “Have you felt sad?”, “Have you felt so bad that you didn't want to do anything?” and “Have you felt lonely?” overfit the model; while in KIDSCREEN-27 parent-proxy version, only the item “Has your child had fun?” under-fit the model. Despite these misfits, when those items were eliminated no improvements on the characteristics of the scale were observed. This result is consistent with reports of Linacre affirming that statistics of fitting between 0.5-1.5 are productive for measuring, those found between 1.5-2.0 are unproductive when it comes to building a measurement, but do not distort it [26].

The validation of KIDSCREEN-27 with a sample of cancer surviving children, reported that items “Have you felt fit and well?” (0.53), “Have you been able to run?” (1.60), “Have your parents treated fairly?” (1.62) and “Have you been able to rely on your friends?” (1.51), misfit the model [10]. Authors explain that those misfits are a consequence of a small sample size, but in our case it could be related to our global assessment of the scale because the original validations and others studies with dimension analysis have reported well goodnes of-fit for all the items when Rasch analysis has been performed [18, 19].

The unidimensionality assessment showed an explained variance close to 50 %, which is an interesting finding given that in this study the scale was analyzed as a whole, without individualizing dimensions. Other studies have performed the analysis for each dimension and have found that explained variance is greater than 50 % [10, 19]. In particular, Jervaesus reports that only the Autonomy & Parent Relations dimension has an explained variance below 50 % (39.8 %), probably because that dimension is a combination of dimensions of KIDSCREEN-52: Autonomy, Relationship with parents and family life, and Economic Resources [10]. Problems related to Autonomy & Parent Relations dimension was also identified and discussed in our validations by classical test theory [20, 21], in which we found that for both KIDSCREEN-27 versions, exploratory factor analysis yielded seven dimensions, unlike the original validation, which has five. After excluding the item “How is in general your health?”, six dimensions were identified. Of these, three correlated with the original dimensions: Physical well-being, Social Support & Peers, and School Environment. But in the case of Autonomy & Parent Relations dimension, items regarding relationships with parents were separated in a different dimension of those regarding money. Confirmatory factor analysis validated the six dimensions found in the exploratory analysis (indices of the model for KIDSCREEN-27 child self-reported version were: CFI = 0.754; NFI = 0.699, and RFI = 0.662; RMSEA = 0.097, GFI = 0.754 and AGFI = 0.701; for the parent-proxy version values were CFI = 0.891, NFI = 0.867, RFI = 0.846; RMSEA = 0.057, GFI = 0.903, and AGFI = 0.88) [20, 21].

Regarding the response form, we found that five categories displayed disordered thresholds between categories 1 and 2 (1 = never/not at all, 2 = seldom/slightly); when these two categories were collapsed in one, separation, reliability, goodness of-fit, and explained variance of the model improved. This finding is consistent with other KIDSCREEN-27 validations that have suggested combining *never* and *seldom* response categories for all items in both versions [10, 19].

One of the strengths of this study was to measure DIF based on age, sex, socioeconomic condition, and health condition. So far there were limited data reporting DIF with KIDSCREEN-27 [10, 22, 33, 34]. DIF was not detected when comparing girls and boys; children (8 to 11 years) and adolescents (12 to 18 years); and ill and healthy kids (except for the item “In general, how would your child rate her/his health?” in parent-proxy version), implying that items of KIDSCREEN-27 are suitable for comparing HRQoL in these Colombian groups. On the other hand, DIF was detected in four items in KIDSCREEN-27 child self-report version and in five items in the parent-proxy version, principally was identified when comparing participants from low and high socioeconomic status. These findings deserve further qualitative research to explain how and why low-income and high-income populations understand these questions differently. DIF would represent a difficulty in the use of KIDSCREEN-27 in Colombia because it means that those items do not measure population independently of their socioeconomic status. However, it is important to consider that performing the whole analysis of the scale, instead of an analysis of dimensions, might taint DIF results since one cause of differential item functioning is the presence of multidimensionality in a test [35].

As this the first research reporting DIF on KIDSCREEN-27, it is important that prospective research assesses DIF so as to establish whether these findings are consistent or specific to the present study.

## Conclusion

KIDSCREEN-27 is a reliable and valid scale to measure HRQoL among Colombian children and adolescents. This validation allows its use in different health contexts in Colombia, for identifying children and adolescents who are at risk for health problems, and planing and evaluating interventions to improve children and adolescents health. Comparisons of HRQoL between kids from low and high income families in Colombia should be interpreted carefully due to differential item functioning (DIF) in four items in the child self-report version, and in five items in parent-proxy version. In the case of the items “Have you had enough money as your friends?” and “Have you had enough money for your expenses?” in KIDSCREEN-27 for parents-proxies, there is a risk of measuring a construct different to the one considered in the dimension Autonomy & Parent Relations. Additionally, it does not measure HRQoL in the same way in families of high and low socioeconomic status. For these reasons, we suggest their inclusion as separate dimensions until there is more data about psychometric properties of these items.
